# Supplementary material for: Rapid and Accurate Detection of Gnomoniopsis smithogilvyi the Causal Agent of Chestnut Rot, through an Internally Controlled Multiplex PCR Assay
Source: Pathogens. 2022 Aug 12;11(8):907. doi: 10.3390/pathogens11080907 (PMC9415963; doi:10.3390/pathogens11080907)
Supplement: Supplementary file 1 [file pathogens-11-00907-s001.zip › Supplementary Figures.pdf]

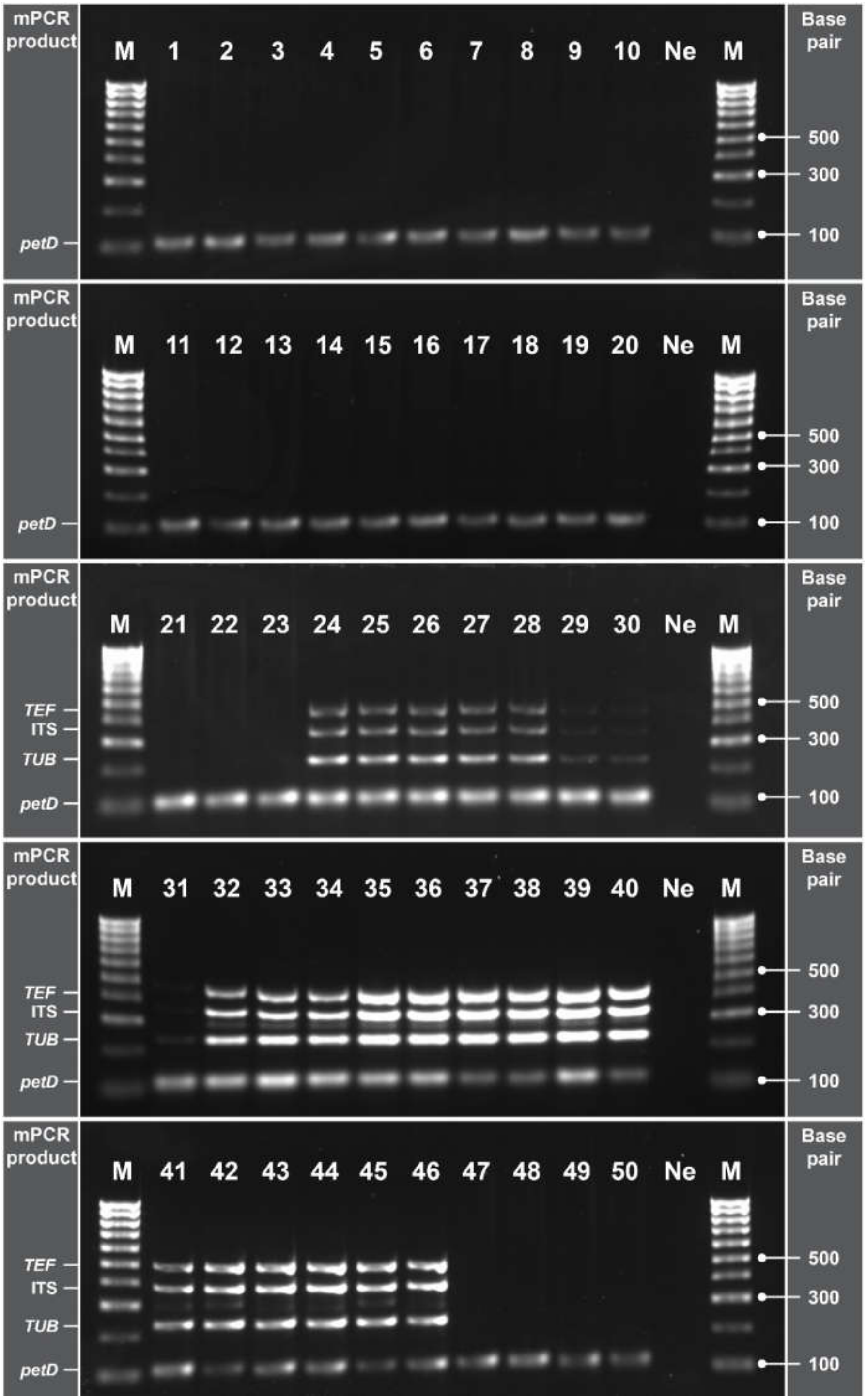

Figure S1. Electrophoretic separation of amplicons derived from *G. smithogilvyi* infected nuts used in mPCR validation (Figure 7). (1-23) symptomless and nut not infected. (24-31) symptomless and infected nuts. (32-46) symptomatic and nuts infected. (47-50) symptomatic and nuts not infected. Lane M: molecular marker (100bp); lane Ne; negative control.

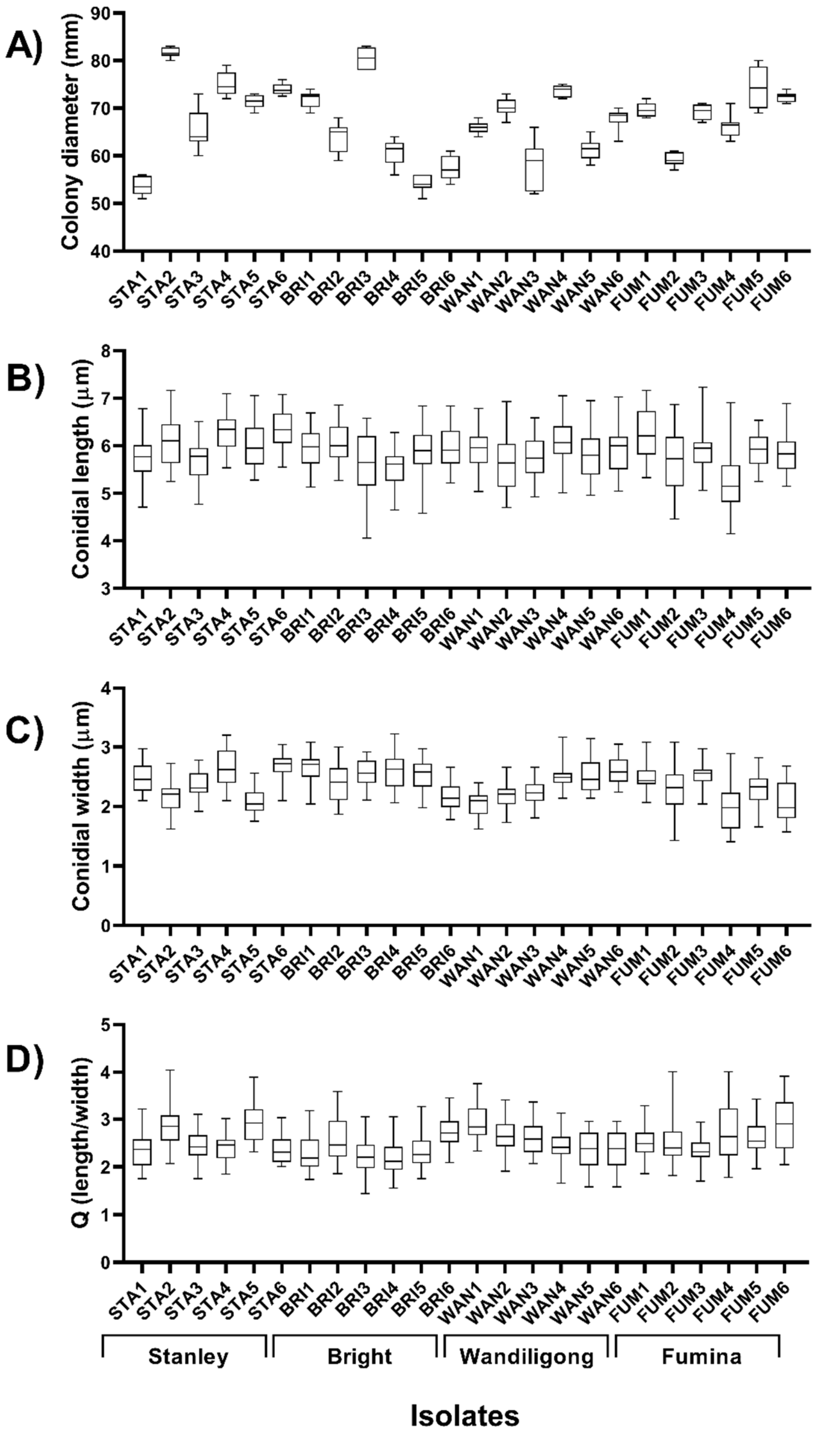

Figure S2. Individual values of the micro and macromorphological characters for each *G. smithogilvyi* isolate. (**A**) Colony size was calculated from 8 plates per isolate. (**B-D**) The conidial size was calculated from 30 conidia per isolate. Morphotypes were sourced from Stanley (STA), Bright (BRI), Wandiligong (WAN), and Fumina (FUM). Box and whisker plots represent the minimum and maximum values of the samples, the line in the middle of the box represents the median.

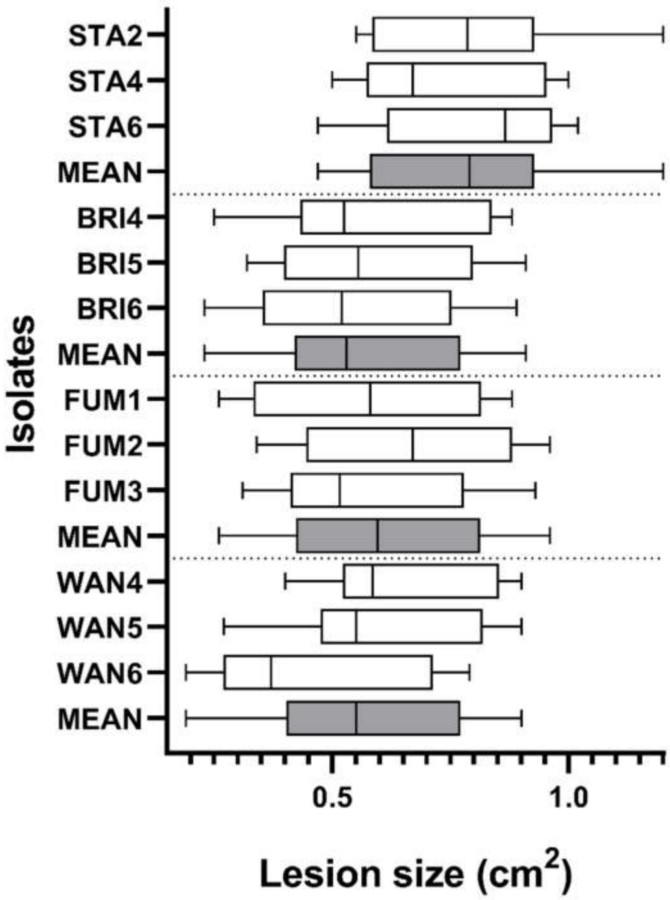

Figure S3. Lesion size caused by selected isolates of *G. smithogilvyi* from each population. Individual values and the mean per group (used in Figure 5) are given. Box and whisker plots represent the minimum and maximum values of the samples, the line in the middle of the box represents the median.
